# Wastewater‐Based Assessment of Antimicrobial Resistance and Bacterial Communities in Urban and Rural Areas in the Province of Trento (Italy)

**DOI:** 10.1002/mbo3.70319

**Published:** 2026-06-01

**Authors:** Maya Petricciuolo, Agnese Carnevali, Alessia Torboli, Mattia Postinghel, Alessia Guasticchi, Paola Foladori, Maria Cadonna, Ermanno Federici

**Affiliations:** ^1^ Laboratory of Applied and Environmental Microbiology, Department of Chemistry, Biology and Biotechnology University of Perugia Perugia Italy; ^2^ Center Agriculture Food and Environment (C3A) University of Trento Trento Italy; ^3^ ADEP Agenzia per la Depurazione (Wastewater Treatment Agency) Trento Italy; ^4^ Department of Civil, Environmental and Mechanical Engineering University of Trento Trento Italy

**Keywords:** antibiotic‐resistance gene, antimicrobial resistance, bacterial community, ESBL‐*E. coli*, urban and rural areas, wastewater‐based epidemiology

## Abstract

Wastewater‐based epidemiology (WBE) can supplement clinical surveillance for assessing the spread of antimicrobial resistance (AMR) across the population. We have analyzed sewage samples from seven wastewater treatment plants in the Province of Trento (Italy) using both culture‐based and metagenomic DNA methods to investigate the prevalence of antimicrobial‐resistant bacteria (ARBs) and resistance genes in urban and rural areas. ESBL‐*Escherichia coli* prevalence was higher in urban areas than in rural ones. As determined by qPCR and dPCR, *intI1* and genes associated with widespread resistances, namely, to tetracyclines (*tetA*), sulfonamides (*sul1*), and fluoroquinolones (*qnrS*), were abundant regardless of the area of origin. Among the genes coding for clinically relevant resistances, only that related to macrolides resistance (*ermB*) was abundant, while the others, namely, those to third‐generation cephalosporins (*bla*_*CTX‐M*_), carbapenems (*bla*_*KPC*_), vancomycin (*vanA*), and methicillin (*mecA*), were detected at much lower concentrations. Further, the abundances of *ermB*, *bla*_*KPC*_, and *vanA* were significantly higher in urban areas. *16S rRNA* amplicon sequencing showed the occurrence of complex bacterial communities and the abundance of *Acinetobacter*, *Pseudomonas*, and *Streptococcus*, genera that may include ARBs reported in the WHO Bacterial Priority Pathogens List, with the latter showing higher prevalence in urban areas. Taken together, our data highlights the importance of implementing WBE studies across geographical areas with different characteristics in terms of vocation, number of municipalities, and population size, such as urban and rural ones. By providing a comprehensive understanding of AMR at the population level, this approach can inform and support more effective public health interventions.

## Graphical abstract

Wastewater from urban and rural areas was analyzed to investigate antimicrobial‐resistant bacteria and resistance genes. ESBL‐*Escherichia coli* and genes coding for clinically relevant resistances, namely, those to macrolides, carbapenems, and vancomycin, were higher in urban than in rural areas.

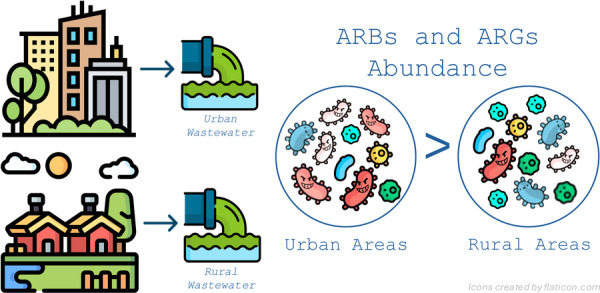

## 1 Introduction

Antimicrobial resistance (AMR), that is, the ability of a microorganism to survive in the presence of an antimicrobial agent, represents one of the major threats to human health, accounting for approximately 1.2 million deaths in 2019 with a foreseen rise to up to 10 million deaths/year by 2050 in the absence of preventive measures (Naghavi et al. 2024). The overuse and misuse of antimicrobial compounds in human medicine, livestock, and agriculture have led to an increased prevalence of antimicrobial‐resistant bacteria (ARBs) and antimicrobial‐resistance genes (ARGs), making infections harder to treat (Holmes et al. 2016; Jian et al. 2021; Alabi et al. 2025). Effective surveillance strategies to monitor AMR prevalence and spread play a key role to face this threat, as they can contribute to implement evidence‐based policies and public health interventions (World Health Organization [WHO] 2025a).

Healthcare‐based surveillance, focusing primarily on specific human pathogens isolated from clinical cases (Hay et al. 2018) provides a partial view of the actual AMR phenomenon because it excludes emerging resistances as well as major carriers of AMR genes, such as commensal and environmental bacteria (Doyle et al. 2025). Wastewater‐based epidemiology (WBE) could provide a comprehensive community‐level view of AMR and is thus regarded as a promising complementary tool to clinical surveillance for rapidly and effectively collecting information on AMR circulations and prevalence (Aarestrup and Woolhouse 2020; Mello et al. 2023; Balcázar 2025). Indeed, surveillance systems to assess viral prevalence and variant circulation through urban sewages were successfully implemented during the COVID‐19 pandemic and remain active even today, pinpointing their potential for monitoring infectious disease threats to human health (Wu et al. 2021; La Rosa et al. 2022; Nappo et al. 2025). Accordingly, the potential of WBE as a resource to face AMR was recently recognized by the revision of the EU Urban Waste Water Treatment Directive (Directive (EU) 2024/3019 2024) and the Italian National Plan for Combating Antimicrobial Resistance (PNCAR 2022–2025) (Italian Ministry of Health 2022), both including permanent AMR surveillance programs based on urban sewage collected at the inlet of municipal wastewater treatment plants (WWTPs).

From a methodological point of view, AMR in sewage can be assessed through culture‐based and metagenomic DNA‐based approaches (Nguyen et al. 2021; Tiwari et al. 2022). Culture‐based methods usually focus on clinically relevant bacterial species, such as those of the ESKAPEE group (*Enterococcus faecium, Staphylococcus aureus, Klebsiella pneumoniae, Acinetobacter baumannii, Pseudomonas aeruginosa, Enterobacter* spp., and *Escherichia coli*) (De Oliveira et al. 2020; Cutrupi et al. 2024). The Tricycle Protocol proposed by the WHO (2025a) for enumerating *E. coli* producing extended‐spectrum β‐lactamases (ESBL‐*Ec*) in human, animal, and environmental samples, including sewages, represents the only standardized procedure available (WHO 2021). Although ESBL‐*Ec* could be a good indicator for AMR risk assessment, a single target provides limited information and cannot provide insights into novel resistances or ARGs transfer. In this respect, the analysis of metagenomic DNA extracted directly from sewage samples allows the simultaneous quantification of several selected ARGs), via quantitative PCR/digital PCR (qPCR/dPCR), or a non‐targeted comprehensive assessment of all the ARGs and ARBs, though next‐generation sequencing (NGS) community profiling (Malcom and Bowes 2025; Punch et al. 2025). Compared with traditional culture‐based methods, these culture‐independent molecular approaches also circumvent the well‐known challenges and biases posed by the inability to culture the predominant fraction of wastewater bacteria, allowing the investigation of AMR in the whole bacterial community rather than only within a limited number of known human pathogens (Miłobedzka et al. 2022).

In Italy, most of the research examining AMR in wastewater has focused on evaluating the efficiency of full‐scale WWTPs or assessing the environmental impact of raw or treated effluent discharged into surface water bodies (Di Cesare et al. 2016; Turolla et al. 2018; Fiorentino et al. 2019; Triggiano et al. 2020; Fonti et al. 2021; Bonetta et al. 2023; Bonanno Ferraro et al. 2024; Gentile et al. 2024). Few studies have used wastewater surveillance to track AMR diffusion within the population and have relied on either culture‐based (Pellegrini et al. 2011; E. Castrignanò, Kannan, et al. 2020; Formenti et al. 2022) or metagenomic‐based (Magnano San Lio et al. 2025) methods. Globally, WBE studies, including those devoted to AMR, have largely focused on urban areas, despite urban–rural health disparities are well documented (Holm et al. 2023). Population density and size have already been identified as factors influencing both the wastewater resistome abundance and composition (Knight et al. 2025), and AMR in sewages from rural communities has been recently investigated (Asaduzzaman et al. 2022; Price et al. 2025). Nevertheless, an actual and punctual comparison of wastewater representing urban and rural areas is still lacking.

In this study, we implemented WBE to investigate the prevalence of ARBs and resistance genes in urban and rural areas through both cultural and metagenomic DNA‐based approaches. Wastewater samples were collected from April 2024 to September 2024 from seven municipal full‐scale WWTPs in the Province of Trento (north‐east of Italy), and the abundances of ESBL‐*Ec* and of selected ARGs, together with the class 1 integron‐integrase (*intI1*) gene, were evaluated. Target ARGs were assessed by qPCR and dPCR and included genes associated with widespread resistances, namely, to tetracyclines (*tetA*), sulfonamides (*sul1*), and fluoroquinolones (*qnrS*), as well as genes involved in clinically relevant resistances, namely, to β‐lactamases (*bla*_*CTX‐M*_ for third‐generation cephalosporin, *bla*_*KPC*_ for carbapenemes, and *mecA* for methicillin), macrolides (*ermB*), and vancomycin (*vanA*). Additionally, the whole bacterial community was characterized by *16S rRNA* amplicon sequencing, focusing on the bacteria listed as known ARBs in the WHO Bacterial Priority Pathogens List (BPPL) (WHO 2024).

## 2 Materials and Methods

### 2.1 Wastewater Sampling and Frequency

A total of 42 raw wastewater samples were collected monthly from April to September 2024 at the inlet of seven WWTPs located in the Province of Trento (north‐east of Italy) after sieving. Refrigerated (4°C) autosamplers were used to form 24‐h composite samples from 96 equal‐volume aliquots per day. Samples were collected in 250‐mL sterile bottles, and two 50 mL aliquots for each sample were immediately sent at refrigerated conditions (4°C) to the University of Perugia for the microbiological analyses carried out within 48 h from the collection. Appendix A Table A1 provides information about the collected sewage, including the sampling point, the sampling date, the flow rate (m^3^/day), the temperature, and the chemical characteristics measured according to ISO 5815‐1:^2019^, ISO 17289:^2014^ for BOD5, and ISO 15923‐1:^2013^ for NH_4_.

### 2.2 Enumeration of ESBL‐*Ec*

A plate count method based on the WHO Tricycle protocol (WHO 2021) was used for the enumeration of ESBL‐*Ec* in all 42 collected sewage samples. Briefly, samples were serial diluted 10, 100, and 1000‐fold in sterile phosphate buffered saline (Euroclone Spa, Pero, MI, Italy) and 100 µL of the undiluted samples, as well as of each dilutions, were plated in duplicate on Tryptone Bile X‐glucuronide agar (TBX‐agar) (VWR International, Milano, Italy) with and without cefotaxime (CTX) 4 µg/mL (Thermo Fisher Scientific, Waltham, MA, USA) followed by 24 h of incubation at 44°C ± 2°C. Then, blue–green colonies growth on TBX‐agar without CTX were enumerated as total *E. coli*, while blue–green colonies growth on TBX‐agar added with CTX were enumerated as ESBL‐*Ec*. The percentage of ESBL‐*Ec* over total *E. coli* was calculated by dividing the number of colony‐forming units (CFUs) of ESBL‐*Ec* by the number of CFUs of total *E. coli*.

### 2.3 Metagenomic DNA Extraction

A volume of 50 mL of each collected sewage sample was concentrated through filtration on sterile polycarbonate filters (0.2 µm porosity) (Sartorius, Goettingen, Germany), followed by metagenomic DNA extraction carried out using the NucleoSpin Soil kit (Macherey‐Nagel, Düren, Germany) following the manufacturer's instructions. Metagenomic DNA samples eluted in 100 µL of sterile elution buffer included in the extraction kit were stored at −20°C.

### 2.4 Quantification of *16S rRNA*, *intI1*, and ARGs Through qPCR and dPCR

A panel of 10 genes was selected for their quantification through conventional qPCR or dPCR. Among them, *16S rRNA* was included for relative abundance calculations, and integron‐integrase *intI1* was included as a marker of anthropogenic pollution and because it is involved in the transfer of ARGs (Keenum et al. 2022). All the other genes are accountable for different resistances, namely, tetracyclines (*tetA*), sulfonamides (*sul1*), fluoroquinolones (*qnrS*), β‐lactamases (*bla*_*CTX‐M*_ for third‐generation cephalosporin, *bla*_*KPC*_ for carbapenemes, and *mecA* for methicillin), macrolides (*ermB*), and vancomycin (*vanA*).

Abundant targets (*16S rRNA*, *sul1*, *tetA*, *qnrS*, and *ermB*) were quantified through qPCR using the LightCycler96 instrument (Roche Diagnostics, Germany). Appendix A Table A2 reports the primer sequences and the PCR conditions used for qPCR assays. In all cases, reactions were performed at a final volume of 20 µL, including 10 μL of Luna Universal qPCR (New England Biolabs, Ipswich, MA, USA), 2 μL of each primer, and 2 μL of a 10‐fold diluted sample. Samples were tested in duplicate, and both positive and negative controls were added to each run. Synthetic oligonucleotides (Eurofins Genomics, Germany), including the targeted sequences, were used as standards to construct calibration curves for absolute quantification using the LightCycler96 Software v1.1.0.1320. Absolute quantification was expressed as gene copies (g.c.) *per* mL of samples (g.c./mL). Further, relative abundances were calculated by normalizing the g.c. of each target for the respective *16S rRNA* g.c.

The less abundant gene targets (*bla*_*CTX‐M*_, *bla*_*KPC*_, *mecA*, and *vanA*) were quantified through dPCR using the QIAcuity Digital PCR System (Qiagen, Hilden, Germany). Appendix A Table A3 reports the primer sequences and the PCR conditions used for dPCR assays, with the exception of *vanA*, which was quantified using a probe‐based commercial assay (DMA00594‐F—dPCR Microbial DNA Detection Assay for *vanA*) (Qiagen, Hilden, Germany) following the manufacturer's instructions. In all the other cases, reactions were performed in a final volume of 12 µL, including 4 μL of 3X EvaGreen PCR Master Mix (FAM channel) (Qiagen, Hilden, Germany), 1.2 μL of each primer, and 2 μL of undiluted sample. QIAcuity 24‐well nanoplates with 8.5K partitions (Qiagen, Hilden, Germany) were used. Negative controls were added to each run together with synthetic oligonucleotides (Eurofins Genomics, Germany), including the targeted sequences used as a positive control. Absolute quantification calculated by the Quicuity Software Suite 2.5.0.1 (Qiagen, Hilden, Germany) was expressed as genomic unit (g.c.) *per* mL of samples (g.c./mL). Further, relative abundances were calculated by normalizing the g.c. of each target for the respective *16S rRNA* g.c.

### 2.5 *16S rRNA* Sequencing and Bioinformatic Analysis

The bacterial *16S rRNA* V5–V6 hypervariable region was amplified using 783F and 1046R primers as previously described (Petroselli et al. 2021). Next‐generation amplicon sequencing was performed at the University of Milano‐Bicocca using the MiSeq Illumina (Illumina Inc., San Diego, CA, USA) with a 2 × 300‐bp paired‐end protocol. Row sequencing data were processed using QIIME2 (v2024.10) (Bolyen et al. 2019) pipeline on CINECA High‐Performance‐Computing platform (T. Castrignanò, Gioiosa, et al. 2020). Sequence filtering, denoising, merging, and chimera elimination were performed using the DADA2 plugin (Callahan et al. 2016). The resulting Amplicon Sequence Variants (ASVs) were classified to the Genus level using the feature‐classifier plugin classify‐sklearn (Pedregosa et al. 2011; Bokulich et al. 2018), referencing the SILVA 138 pretrained classifier Quast et al. 2013; Robeson et al. 2021).

### 2.6 Statistical Analyses

Analyses were performed with R Studio (v2025.09.0‐387). Check of distribution and scedasticity of data, data summary and statistical analysis (Dunn's test and Wilcoxon rank‐sum test) were conducted through *rstatix* v0.7.3 package (Kassambara 2025a), while boxplots were generated using *ggplot2* v4.0.1 (Wickham 2016), *ggpublr* v0.6.2 (Kassambara 2025b) and *scales* v1.4.0 (Wickham et al. 2025) packages. Spearman's correlation coefficients were calculated using the base stats package (cor and cor.test functions) and visualized using the corrplot v0.95 package (Wei and Simko 2024), including only correlation coefficients with *p* < 0.05.

As for microbiota, *phyloseq* v1.54.0 (McMurdie and Holmes 2013), *vegan* v2.7‐2 (Oksanen et al. 2025), and *ggplot2* v4.0.1 packages were used for analysis and for graph generation. Only ASVs with at least 0.5% counts across all samples were retained and used for alpha diversity (Shannon's metrics) and beta diversity (Bray–Curtis dissimilarity and Weighted UniFrac) calculation. PERMANOVA and pairwise‐adonis were carried out through *vegan* v2.7‐2 and *pairwiseAdonis* v0.4.1 (Martinez 2017) packages. Differential abundance analysis on *phyla* and *genera* abundance was carried out using the *DESeq. 2* v1.50.2 package with Wald test (Love et al. 2014) after grouping ASVs to a select taxonomic levels and including in the analyses only the most abundant phyla (> 3%) and genera (> 1%). Results with an adjusted (Benjamini and Hochberg method) *p* ≤ 0.05 and log2fold > 1 were considered significant.

## 3 Results

### 3.1 Wastewater as a Representative of Rural and Urban Areas

**Figure 1:**
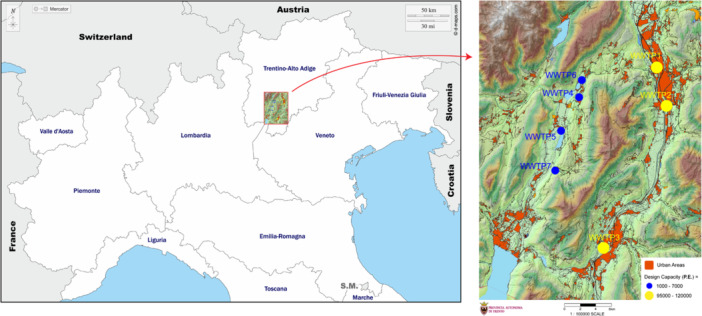
Map showing the locations of the selected WWTPs. Urbanized areas are represented in red. Yellow dots represent WWTPs with a design capacity of 95,000–120,000 P.E. serving urban areas (i.e., WWTP1, 2, and 3), whereas blue dots represent WWTPs with a design capacity of 1000–7000 P.E. serving rural areas (i.e., WWTP4, 5, 6, and 7). Modified from the Physical Map of Trentino available from WebGIS PAT (https://webgis.provincia.tn.it/), Provincia Autonoma di Trento (accessed on March 27, 2026). P.E., Population Equivalent; WWTP, wastewater treatment plant.

Wastewater samples were collected from seven WWTPs in the Province of Trento (north‐east of Italy). Despite located in a relatively limited territory (i.e., 6207 km^2^), as shown by the map reported in Figure 1, the WWTPs can be regarded as representative of geographical areas characterized by different vocations, number of municipalities, and population size (Table 1). Each sewershed is, in fact, comprised of an independent sewerage system, operating separately from the stormwater drainage infrastructure. Consequently, municipal wastewater is mainly influenced by residential, commercial, institutional, recreational, and industrial activities, with limited or no influence from agricultural practices, stormwater, or hauled wastewater. On the basis of the vocation of the geographical areas, two distinct groups could be identified, namely, an urban one (served by WWTP1, 2, and 3) and a rural one (served by WWTP4, 5, 6, and 7). In addition, two of the urban areas, specifically those served by WWTP2 and WWTP3, include hospitals: 4 with a total of 852 beds in the former and 2 with a total of 367 beds in the latter.

**Table 1:** Characteristics of the geographical areas served by the selected WWTPs, with different parameters used to express the size of the served population.

Site acronym	Vocation of the served area	No. (or part of) municipalities	Census population (inh.)	WWTP design capacity (P.E.)	Mean P.E. based on BOD5 (sd)	Mean P.E. based on NH_4_ (sd)
WWTP1	Urban	6	86,000	120,000	76,730 (20,830)	92,326 (8946)
WWTP2	Urban	1	47,000	100,000	62,943 (15,308)	50,353 (7714)
WWTP3	Urban	9	55,000	95,000	58,716 (16,131)	50,670 (7266)
WWTP4	Rural	3	2800	7000	4395 (1707)	2765 (1017)
WWTP5	Rural	4	4400	5000	1641 (263)	1161 (430)
WWTP6	Rural	2	3000	3500	2394 (769)	1139 (349)
WWTP7	Rural	2	1800	1000	1177 (783)	545 (253)

Abbreviations: inh., inhabitants; P.E., Population Equivalent; WWTP, wastewater treatment plant.

The two groups of geographical areas identified were also characterized by very different population sizes. This was independent of the parameter used, whether it was the census population, that is, the total resident inhabitants (inh.) connected to the WWTP, the Population Equivalent (P.E.) determined by the WWTP's design capacity, or the P.E. calculated based on either BOD5 values or NH_4_ levels. The census population varied from 1800 to 86,000 inh., while the P.E. based on WWTP design capacity ranged from 1000 to 120,000 P.E. While these two parameters are fixed values, the punctual P.E. values can be daily calculated considering the sewershed load effectively produced and reaching the WWTPs, as determined by BOD5 or ammonium nitrogen (NH_4_). Throughout this study, we have considered P.E. based on BOD5 as the most representative of the actual size of the population served by each WWTP, due to its temporal variability reflecting population fluctuations. Value of this parameter ranged from 1177 ± 783 P.E. for the smallest area (served by WWTP7) to 76,730 ± 20,830 P.E. for the largest (served by WWTP1) and showed an approximate 10‐fold difference between the largest treatment plant serving rural areas (4395 ± 1707 P.E. for WWTP4) and the smallest one serving urban areas (58,716 ± 16,131 for WWTP3). Consequently, the two groups of geographical areas could also be regarded as those served by WWTPs with P.E. based on BOD5 > 50,000 (urban) and P.E. < 5000 (rural).

### 3.2 Prevalence of ESBL‐*Ec*

**Figure 2:**
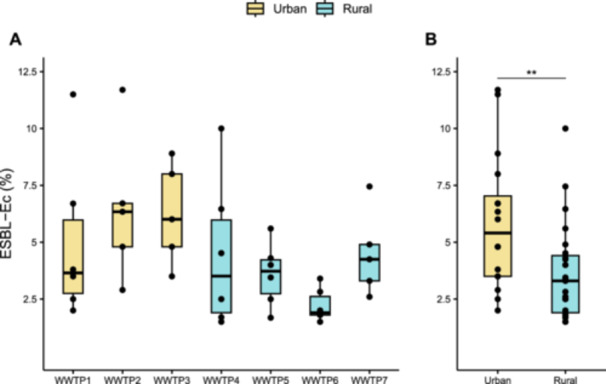
Comparative analysis of ESBL‐*Ec* prevalence. Boxplot shows the percentage of ESBL‐*Ec* over total *Escherichia coli* by WWTP (A) and by groups of geographical areas (B). Yellow boxes represent WWTPs serving urban areas (i.e., WWTP1, 2, and 3), whereas blue boxes represent WWTPs serving rural areas (i.e., WWTP4, 5, 6, and 7). Box represents the 25%–75% quartile range and the solid line within each box indicated the median. Significant p value (*p* < 0.05) from Dunn's test (A) and from Wilcoxon rank‐sum test (B) are reported (*p < 0.05; **p < 0.01; ***p < 0.001; ****p < 0.0001).

Total *E. coli* and extended‐spectrum β‐lactamases producing *E. coli* (ESBL‐*Ec*) in sewage samples were enumerated by cultural analysis (Figure 1 and Table B1). *E. coli* and ESBL‐*Ec* were detectable in all samples with a median load value of 2.72 × 10^6^ CFU/100 mL (interquartile range [IQR] = 2.21 × 10^6^) and 9.75 × 10^4^ CFU/100 mL (IQR = 8.5 × 10^4^), respectively. The prevalence of ESBL‐*Ec*, expressed as a percentage over total *E. coli* for wastewater samples from each geographical area, is shown in Figure 2A. Overall, the average median value was 3.8% (IQR = 3.68%), with samples from WWTP2 and WWTP3, representing densely urbanized areas which include hospitals, showing the highest median values (6.34% and 6.01%, respectively) and samples from WWTP6, corresponding to a small rural area, with the lowest value (1.90%). Aggregating data into two groups of geographical areas demonstrated that ESBL‐*Ec* prevalence was significantly higher (Wilcoxon rank‐sum test, *p* < 0.01) in urban (5.41%) than rural (3.30%) areas (Figure 2B).

### 3.3 Abundance of ARGs and *intI1*

Absolute abundances of ARGs (i.e., *sul1, tetA, ermB, qnrS, bla*_*CTX‐M*_, *bla*_*KPC*_, *mecA*, and *vanA*) and *intI1*, as a marker of anthropogenic pollution and gene transfer, were quantified by qPCR and dPCR analysis, and relative abundances computed with *16S rRNA* gene as a normalizer (Table 2). Overall, mean absolute and relative abundances evidenced that *intI1* was the most abundant gene, followed by *sul1* > *ermB* > *qnrS* > *tetA* > *bla*_*CTX‐M*_ > *bla*_*KPC*_ > *vanA ~mecA*.

**Table 2:** Target gene concentrations in wastewater samples. For each gene, absolute (gene copies (g.c.)/mL) and relative (target gene g.c./*16S rRNA* gene g.c.) abundances are reported as minimum (min) and maximum (max) values, mean with standard deviation (sd), and median with interquartile range (IQR).

	Absolute abundance (g.c./mL)			Relative abundance (target g.c./*16S rRNA* g.c.)		
	Minimum–Maximum	Mean (sd)	Median (IQR)	Minimum–Maximum	Mean (sd)	Median (IQR)
*16S rRNA*	1.70 × 10^7^–6.82 × 10^8^	2.22 × 10^8^ (1.54 × 10^8^)	1.54 × 10^8^ (2.14 × 10)			
*intI1*	1.85 × 10^5^–6.11 × 10^6^	1.89 × 10^6^ (1.85 × 10^6^)	1.46 × 10^6^ (1.72 × 10^6^)	4.03 × 10^−^^3^–2.11 × 10^−^^2^	4.03 × 10^−^^3^ (2.11 × 10^−^^2^)	8.95 × 10^−^^3^ (5.36 × 10^−^^3^)
*sul1*	1.18 × 10^5^–4.81 × 10^6^	1.31 × 10^6^ (9.77 × 10^5^)	9.64 × 10^5^ (1.02 × 10^6^)	2.25 × 10^−^^2^–2.02 × 10^−^^2^	4.47 × 10^−^^3^ (3.21 × 10^−^^3^)	6.12 × 10^−^^3^ (3.40 × 10^−^^3^)
*ermB*	3.28 × 10^4^–3.77 × 10^6^	6.67 × 10^5^ (6.32 × 10^5^)	5.45 × 10^5^ (5.62 × 10^5^)	4.49 × 10^−^^4^–1.15 × 10^−^^2^	3.17 × 10^−^^3^ (2.16 × 10^−^^3^)	2.44 × 10^−^^3^ (2.03 × 10^−^^3^)
*qnrS*	1.60 × 10^3^–2.05 × 10^6^	3.56 × 10^5^ (4.65 × 10^5^)	1.19 × 10^5^ (3.51 × 10^5^)	9.41 × 10^−^^5^–1.42 × 10^−^^2^	1.51 × 10^−^^3^ (2.32 × 10^−^^3^)	8.43 × 10^−^^4^ (1.18 × 10^−^^3^)
*tetA*	1.21 × 10^4^–7.33 × 10^5^	1.81 × 10^5^ (1.32 × 10^5^)	1.50 × 10^5^ (1.48 × 10^5^)	4.62 × 10^−^^4^–2.81 × 10^−^^3^	8.82 × 10^−^^4^ (4.22 × 10^−^^4^)	7.58 × 10^−^^4^ (3.96 × 10^−^^4^)
*bla*_*CTX‐M*_	3.61 × 10^1^–3.55 × 10^5^	1.26 × 10^4^ (5.43 × 10^4^)	2.92 × 10^3^ (4.73 × 10^3^)	2.21 × 10^−^^6^–7.02 × 10^−^^4^	3.77 × 10^−^^5^ (1.07 × 10^−^^4^)	1.47 × 10^−^^5^ (1.52 × 10^−^^5^)
*bla*_*KPC*_	0.00–1.49 × 10^4^	1.35 × 10^3^ (2.87 × 10^3^)	6.58 × 10^1^ (1.37 × 10^3^)	0.00–4.63 × 10^−^^5^	5.56 × 10^−^^6^ (1.11 × 10^−^^5^)	3.62 × 10^−^^7^ (4.43 × 10^−^^6^)
*vanA*	0.00–8.53 × 10^2^	1.09 × 10^2^ (2.21 × 10^2^)	8.14 (5.48 × 10^1^)	0.00–4.87 × 10^−^^6^	4.70 × 10^−^^7^ (1.00 × 10^−^^6^)	6.19 × 10^−^^8^ (3.62 × 10^−^^7^)
*mecA*	8.67–2.05 × 10^2^	7.83 × 10^1^ (4.33 × 10^1^)	7.32 × 10^1^ (6.04 × 10^1^)	1.00 × 10^−^^7^–1.72 × 10^−^^6^	4.71 × 10^−^^7^ (3.41 × 10^−^^7^)	3.71 × 10^−^^7^ (4.25 × 10^−^^7^)

**Figure 3:**
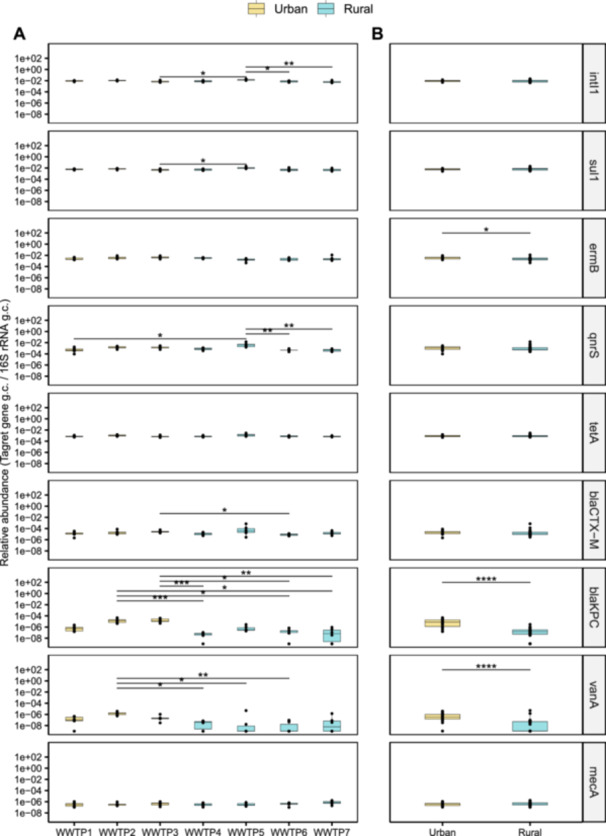
Comparative analysis of target gene abundances. Boxplot shows the relative abundances of target genes, normalized to *16S rRNA*, by WWTP (A) and by groups of geographical areas (B). Yellow boxes represent WWTPs serving urban areas (i.e., WWTP1, 2, and 3), whereas blue boxes represent WWTPs serving rural areas (i.e., WWTP4, 5, 6, and 7). The box represents the 25%–75% quartile range, and the solid line within each box indicates the median. Significant p value (*p* < 0.05) from Dunn's test (A) and from Wilcoxon rank‐sum test (B) are reported (*p < 0.05; **p < 0.01; ***p < 0.001; ****p < 0.0001). ESBL, extended‐spectrum β‐lactamases; WWTP, wastewater treatment plant.

Figure 3A shows the relative abundances of target genes in wastewater samples from each geographical area. All genes were found in at least one sample from each site but, while *intI1* and most ARGs were always detected, *bla*_*KPC*_ and *vanA* genes were found in only 90.5% and 66.7% of samples, respectively. Interestingly, most of negative samples for *bla*_*KPC*_ and *vanA* were collected from WWTP serving rural areas, whereas those from urban areas were always positive for both genes with the exception for one sample from Site‐1 resulted negative for *vanA*. When comparing different sites, the highest number of significant differences (Dunn's test with Bonferroni correction, *p* < 0.05) were observed for *bla*_*KPC*_ and, to a lesser extent, for *intI1, sul1, qnrS, bla*_*CTX‐M*_, and *vanA*. Considering the two main groups of geographical areas, the relative abundances of *bla*_*KPC*_, *vanA*, and *ermB* were significantly higher (Wilcoxon rank‐sum test, *p* < 0.0001) in urban than rural areas (Figure 3B).

**Figure 4:**
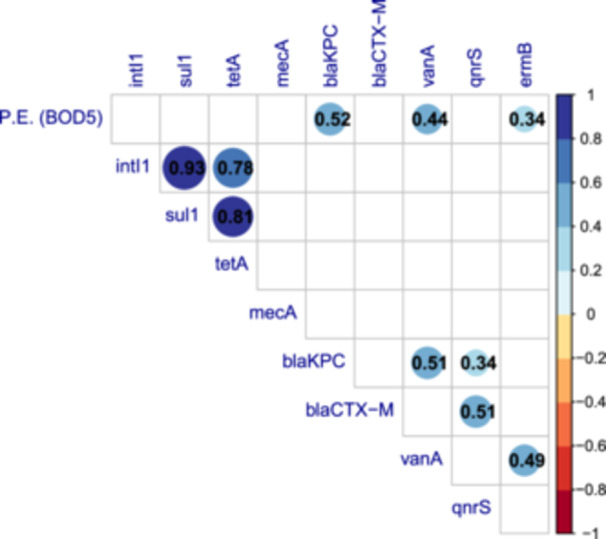
Spearman's correlation matrix among target genes and between target genes and population size. Relative abundances of target genes, normalized to *16S rRNA*, and population size estimated as P.E. based on BOD5, have been considered. Only statistically significant (*p* < 0.05) Spearman's correlations are shown in the matrix, with blue and red circles indicating positive and negative correlations, respectively. Color intensity and the size of the circles are proportional to Spearman's correlation coefficients (*ρ*), also reported as a black number within each circle. White spaces indicate no significant correlation. BOD, biochemical oxygen demand; P.E., Population Equivalent.

To assess possible associations among genes, and between genes and population size, estimated as P.E. based on BOD5, Spearman's correlation matrix was calculated (Figure 4). As expected, the relative abundances of *bla*_*KPC*_, *vanA*, and *ermB* showed significant (Spearman's correlation, *p* < 0.05) positive correlations with population size. Among genes, strong positive correlations were observed between *intI1*, *sul1* and *tetA*. Weaker, although significant, positive correlations were observed for both *bla*_*KPC*_ and *bla*_*CTX‐M*_ with *qnrS*, and for *vanA* with *bla*_*KPC*_ and *ermB*.

**Figure 5:**
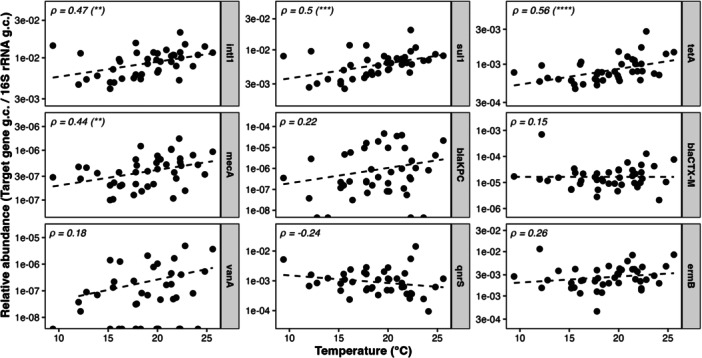
Correlations between target genes and wastewater sample temperature. Each point represents values of relative gene abundance, normalized to the *16S rRNA* gene, and temperature of a single wastewater sample. Dashed lines indicate linear regression trends. Spearman's correlation coefficients (*ρ*) were reported within each plot together with the *p* value (*p < 0.05; **p < 0.01; ***p < 0.001; ****p < 0.0001).

Figure 5 shows the relationship between the relative abundance of ARGs and wastewater sample temperature. Most genes showed an increasing trend associated with wastewater sample temperature rise, whereas significant correlation only occurred for *intI1*, *sul1*, *tetA*, and *mecA*.

### 3.4 Bacterial Community Characterization

**Figure B1:**
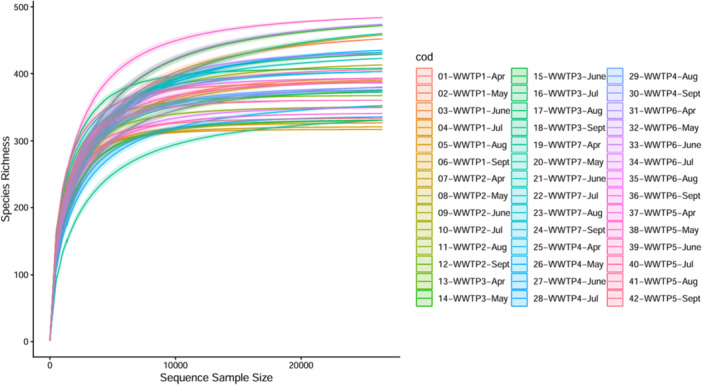
Rarefaction curve. The plot shows the rarefaction curves for each sample, calculated after rarefaction to 26.500 reads. WWTP, wastewater treatment plant.

**Figure B2:**
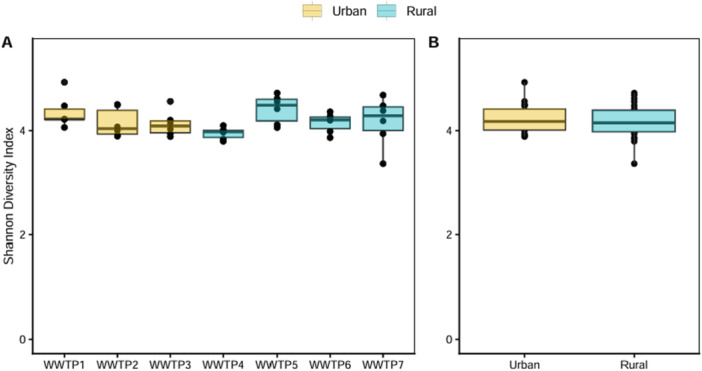
Comparative analysis of alpha diversity. Boxplots show the alpha diversity (Shannon Index) by WWTP (A) and by geographic area groups (B). Yellow boxes represent WWTPs serving urban areas (i.e., WWTP1, 2, and 3) whereas blue boxes represent WWTPs serving rural areas (i.e., WWTP4, 5, 6, and 7). Box represents the 25%–75% quartile range and the solid line within each box indicated the median. WWTP, wastewater treatment plant.

**Figure B3:**
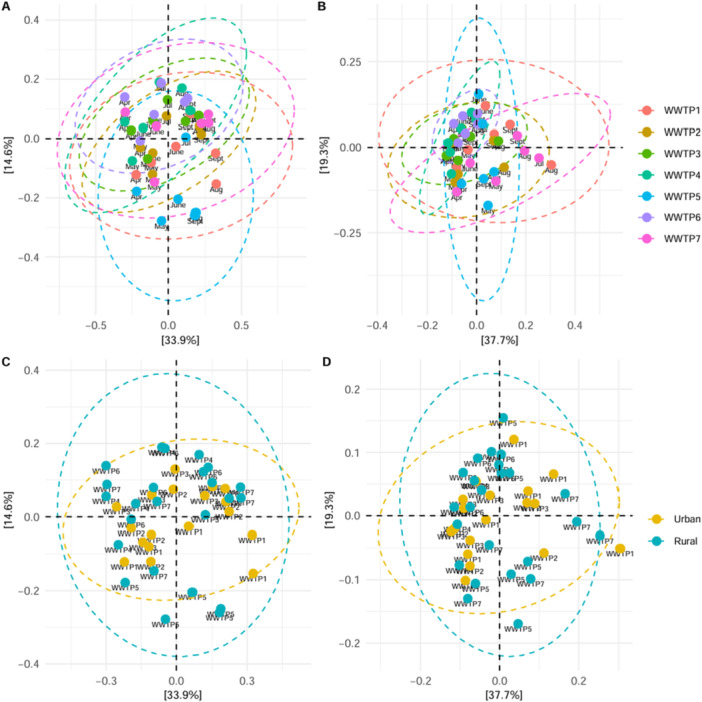
Beta‐diversity analysis. Bray–Curtis dissimilarity (A–C) and Weighted UniFrac distance (B–D) were represented via principal coordinates analysis (PCoA) to compare the sample distribution between WWTPs (A, B) and geographical areas (C, D). WWTP, wastewater treatment plant.

The structure and composition of the whole bacterial community present in wastewater samples were characterized by Illumina NGS analysis of the V5–V6 hypervariable regions of the *16S rRNA* gene. A minimum of 28,373 to a maximum of 453,704 high‐quality *non*‐chimeric sequences *per* sample were obtained, and a total of 7326 ASVs were identified. After removing *non*‐bacterial sequences and rare ASVs (i.e., those < 0.5% of the total sequences), each sample was rarefied to 26,500 reads. Rarefied table accounted for 1,113,000 total sequences and 576 ASVs, which described the whole bacterial diversity present in each sample, as evidenced by the rarefaction curves reaching the plateau (Figure B1). Comparing the α‐diversity indexes calculated using Shannon's metrics to estimate the community diversity in each sample, no significant differences were observed among WWTPs serving the different geographical areas or between the urban and rural areas (Figure B2). Analysis of β‐diversity was carried out by computing Bray–Curtis dissimilarity and Weighted UniFrac distances to assess possible differences in bacterial community composition (Figure B3). No significant differences were observed among the bacterial communities of samples from the different WWTPs (pairwise‐PERMANOVA), except for a unique significant difference (*p* < 0.042) among the centroids of WWTP6 and WWTP7. Significant differences in bacterial communities were not found when comparing the two groups of WWTPs serving urban and rural areas.

**Figure 6:**
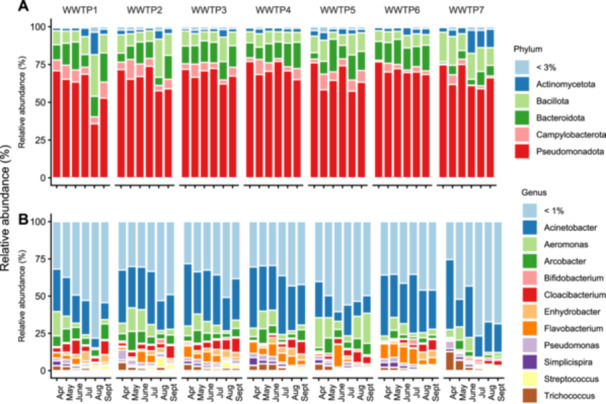
Stacked bar plot showing bacterial community composition. Bar plots report relative abundances of the most abundant bacterial phyla (A) and genera (B) in WWTPs monitored monthly from April 2024 to September 2024. Taxonomy was assigned based on the SILVA‐138 database. Only phyla with a relative abundance > 3% and genera with a relative abundance > 1% are reported. WWTP, wastewater treatment plant.

Taxonomic analysis was carried out to identify bacterial populations. Overall, 94.61% of the sequences and 96.53% of the ASVs were classified at the phylum level, while 78.45% of the sequences and 71.18% of the ASVs were classified at the genus level. The most abundant phyla (> 3%) were Pseudomonadota (35.75%–76.97% and mean = 66.87%), Bacillota (4.69%–27.57% and mean = 10.75%), Bacteroidota (1.34%–21.55% and mean = 11.73%), Campylobacterota (0.40%–13.0% and mean = 5.12%), and Actinomycetota (1.44%–15.26% and mean = 4.01%) (Figure 6A). Despite a total of 152 different genera were identified, only 10 of them exceeded the average relative abundance of 1% (Figure 6B), namely, *Acinetobacter* (6.49%–47.0% and mean = 26.33%), *Aeromonas* (0.74%–20.28% and mean = 7.05%), *Arcobacter* (0.4%–12.97% and mean = 5.10%), *Flavobacterium* (0.26%–15.10% and mean = 4.06%), *Cloacibacterium* (0.10%–9.75% and mean = 3.85%), *Trichococcus* (0.48%–12.63% and mean = 2.20%), *Enhydrobacter* (0.08%–6.29% and mean = 2.06%), *Pseudomonas* (0.19%–6.71% and mean = 1.92%), *Simplicispira* (0.10%–5.18% and mean = 1.34%) and *Streptococcus* (0.15%–6.73% and mean = 1.08%). Notably, among the most abundant genera, only *Streptococcus* exhibited statistically significant differences in abundance between WWTPs serving urban and rural areas (Table B2).

### 3.5 Abundance of Bacterial Priority Pathogens

**Figure 7:**
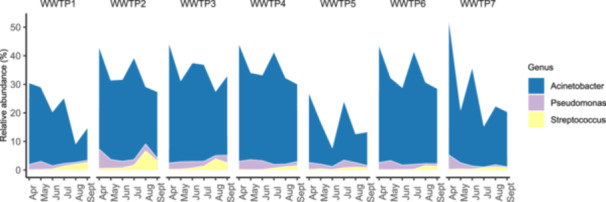
Temporal trend of *Acinetobacter*, *Pseudomonas*, and *Streptococcus* abundance. A stacked bar plot shows the relative abundance over time of the three abundant genera (relative abundance > 1%) putatively comprising species listed in the WHO Bacterial Priority Pathogens List 2024 (BPPL 2024). Each plot represents a single WWTP monitored monthly from April 2024 to September 2024. ESBL, extended‐spectrum β‐lactamases; WHO, World Health Organization; WWTP, wastewater treatment plant.

Among the 10 genera exceeding the average relative abundance of 1%, *Acinetobacter*, *Pseudomonas*, and *Streptococcus* included species listed in the WHO Bacterial Priority Pathogens List 2024 (BPPL 2024) because are known as ARBs (WHO 2024). In most of the sites, *Acinetobacter* and *Pseudomonas* relative abundance decreased over time, while *Streptococcus* increased (Figure 7 and Table B3). Considering the average among sites, *Acinetobacter* decreased from 37.04% in April to 20.98% in September and *Pseudomonas* from 3.34% to 1.09%, while *Streptococcus* rose from 0.28% to 1.88%.

**Figure 8:**
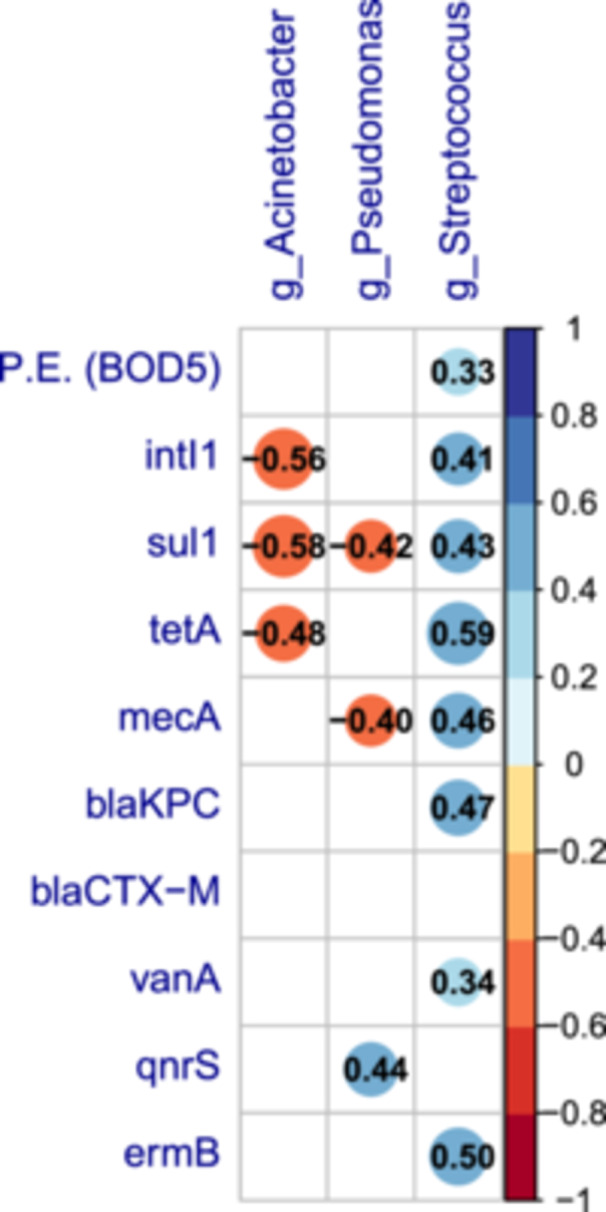
Spearman's correlation matrix showing the co‐occurrence of bacteria listed in the BPPL 2024 and the relative abundances of targeted genes. The correlation matrix shows only statistically significant (*p* < 0.05) Spearman's correlations. White spaces indicate no significant correlation. Blue circles and red circles indicated positive and negative correlations, respectively. The color and the size of the circle are proportional to Spearman's correlation coefficients (*ρ*) also reported as a black number within each circle. BPPL 2024, Bacterial Priority Pathogens List 2024; WWTP, wastewater treatment plant.

To assess a possible association between the abundances of these three genera and the population size (P.E. based on BOD5), as well as with the abundances of target genes, Spearman's correlation analysis was carried out (Figure 8). Consistent with the observed higher abundance of *Streptococcus* in samples from urban areas, this genus showed a significant, although weak, positive association with population size. Furthermore, *Streptococcus* positively correlated with *intI1* and several ARGs, namely, *sul1, tetA, mecA, blaKPC, ermB*, and *vanA*. In contrast, *Pseudomonas* and *Acinetobacter* showed fewer, predominantly negative, correlations (the former with *sul1* and *mecA*, and the latter with *intI1*, *sul1*, and *tetA*). The only positive association noted was between *qnrS* and *Pseudomonas*.

**Figure 9:**
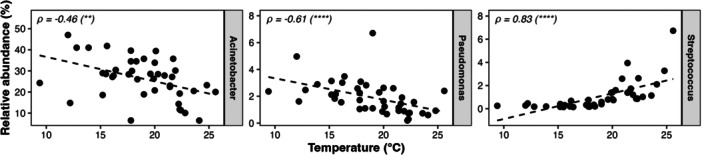
Correlations between *Acinetobacter*, *Pseudomonas*, and *Streptococcus* abundance and wastewater sample temperature. Each point represents the relative abundance of the three most abundant genera (relative abundance > 1%) putatively comprising species listed on the WHO Bacterial Priority Pathogens List 2024 (BPPL 2024) and the temperature of a single wastewater sample. Dashed lines indicate linear regression trends. Spearman's correlation coefficients (*ρ*) were reported within each plot together with the *p* value (*p < 0.05; **p < 0.01; ***p < 0.001; ****p < 0.0001).

Regarding the wastewater sample temperature, a strong positive correlation was observed for *Streptococcus* (Spearman's correlation, *ρ* = 0.86), while *Acinetobacter* and *Pseudomonas* showed negative correlations (Spearman's correlation, *ρ* = −0.46 and −0.61, respectively) (Figure 9).

## 4 Discussion

AMR is considered among the top 10 global threats to human health (WHO 2025b). Despite AMR in bacteria naturally occurring as an evolutionary strategy to survive to antimicrobial products synthetized by other microorganisms (D'Costa et al. 2011), the extensive use of antibiotics in human and veterinary medicine, as well as in livestock and agriculture, is leading to a continuous rise in the prevalence of ARBs and ARGs prevalence making infections harder to treat (Holmes et al. 2016; Jian et al. 2021). Monitoring strategies to integrate data from clinical and veterinary surveillance, providing a population‐level view of ARBs and ARGs circulation and supporting early detection of resistance, are needed. In these respects, WBE is regarded as a promising complementary tool for rapidly and effectively collecting information on AMR circulations and prevalence (Aarestrup and Woolhouse 2020; Balcázar 2025).

In line with the WBE approach, in this work, we have investigated the prevalence of ARBs and ARGs in urban and rural areas of the Province of Trento (north‐east of Italy) by applying cultural and molecular analyses of wastewater samples. In the last few years, several studies have used wastewater surveillance to unravel AMR diffusion and trends in human populations, as recently reviewed (Malcom and Bowes 2025; Punch et al. 2025), but very few have been carried out in Italy and only using culture‐based (Pellegrini et al. 2011; E. Castrignanò, Kannan, et al. 2020; Formenti et al. 2022) or metagenomic‐based (Magnano San Lio et al. 2025) approaches separately. We have integrated both approaches in the same study using cultures to evaluate the prevalence of Extended‐Spectrum β‐Lactamase‐producing *E. coli* (ESBL‐*Ec*), qPCR/dPCR to assess the abundance of selected ARGs, and *16S rRNA* amplicon sequencing to characterize the whole bacterial community and identify bacterial priority pathogens. Despite the WWTPs monitored in this study are all localized in the same province, each WWTP is included in a distinct sewershed with an independent sewerage system and serves a geographical area well characterized in terms of vocation, number of municipalities and population size. This information enabled us to distinctly separate the wastewater samples into two groups: those representing urban areas and those representing rural areas. Wastewater‐based monitoring of pathogens or health markers has largely focused on urban areas, despite urban–rural health disparities being well documented (Holm et al. 2023). Recently, ARBs and ARGs have been investigated in wastewater from rural communities (Asaduzzaman et al. 2022; Price et al. 2025), but an actual and punctual comparison of wastewater representing urban and rural areas is still lacking.

As expected, the quantification of *E. coli* strain producing extended‐spectrum β‐lactamases (ESBL‐*Ec*) revealed that ESBL resistance is widely spread in the studied area with an overall median value of 3.8% (IQR = 3.68%), consistent with a previous survey reporting a similar ESBL‐*Ec* prevalence in Italy (3.2%) and in accordance with results of other European countries, such as Denmark (3.6%), Finland (3.6%), and France (3.2%) (Huijbers et al. 2020). Our data also showed that urban areas, especially those with hospitals, had a higher prevalence of ESBL‐*Ec* than rural areas. In agreement with previous publications reporting similar trends in Germany and Switzerland (Schmiege et al. 2021; Conforti et al. 2024), this evidence suggests that urban areas may facilitate ESBL‐*Ec* transmission due to a higher population density and to the presence of hospitals known to have higher ARB rates compared with community settings (Lan et al. 2025).

Despite the prevalence of ESBL‐*Ec* representing a valuable proxy for AMR trend (WHO 2021), a single parameter associated with a unique resistance provides limited information. We have thus used qPCR and dPCR to quantify eight ARGs, together with *intI1* as a marker of anthropogenic impact and because of its role in horizontal gene transfer (HGT) of ARGs (Di Cesare et al. 2016). PCR‐based methods overcome challenges and biases associated with culturing, providing a reproducible and highly sensitive measure of ARGs across the entire bacterial community and not only of those carried by known pathogens (Miłobedzka et al. 2022). Target ARGs included those linked to resistance against sulfonamides (*sul1*) and tetracyclines (*tetA*), which are common in human pathogens and widespread in the environment because of their long and extensive use in human and veterinary medicine (Liguori et al. 2022). We have also included *qnrS*, accountable for plasmid‐related resistance to low levels of fluoroquinolones, due to its known presence in the water environment and WWTPs, and as an indicator of anthropogenic input (Abramova et al. 2023). These antibiotics, in fact, are mostly *non*‐metabolized by the human body and so are excreted as active compounds that can induce resistance in environmental bacteria due to HGT phenomena (E. Castrignanò, Yang, et al. 2020). We have also quantified ARGs for their involvement in clinically relevant resistances, namely, *ermB*, *bla*_*CTX‐M*_, *bla*_*KPC*_, *mecA*, and *vanA*. *erm* gene classes are frequently found in macrolide‐resistant bacterial strains, whose clinical relevance is mostly associated with gram‐positive pathogens, such as *Streptoccoccus* (Nor Amdan et al. 2024), with *ermB* conferring resistance to high antibiotic levels and its presence leading to the macrolides, lincosamides, and streptogramin B (MLS) resistant phenotype (Harimaya et al. 2007). *bla*_*CTX‐M*_ and *bla*_*KPC*_, as well as *mecA*, are responsible for β‐lactams resistance. However, each gene product targets a different class of β‐lactams. The Ambler Class A Serine‐ β‐lactamase codified by *bla*_*CTX‐M*_ has replaced over the last few years the sulfhydryl variable and temoneira genes as the main ESBL enzyme associated with penicillin and third‐generation cephalosporins resistance and frequently occurs in clinical *E. coli* strains non‐responding to β‐lactams treatment (Castanheira et al. 2021). *bla*_*KPC*_ carbapenemase product is also included in the Ambler Class A Serine β‐lactamase group, and increasingly occurs in carbapenem‐resistant Enterobacteriaceae infection, especially those caused by *K. pneumoniae* and *E. coli* (Chen et al. 2014). Instead, *mecA* codified for a penicillin‐binding protein PBP 2A possess low affinity for penicillin and penicillin like drugs together with methicillin, and its clinical relevance is mostly associated with methicillin‐resistant *S. aureus* (MRSA) strains (Wielders et al. 2002). A *vanA* gene leads to glycopeptides resistance and mostly occurs in vancomycin‐resistant *Enterococci* (VRE), such as *E. faecium* and *fecalis* (Guzman Prieto et al. 2016).

Overall, *intI1* was the most abundant gene in all geographical areas, followed by those ARGs known as widespread in the environment and human pathogens, and as an indicator of anthropogenic input, that is, *sul1*, *tetA* and *qnrS*. All these genes showed mean absolute abundances ranging from 10^5^ to 10^6 ^g.c./mL with few differences in relative abundance between sites, and similar values in WWTPs serving urban and rural areas, confirming their high prevalence in environmental bacteria and commensal microbiota as already observed in other surveys (Abramova et al. 2023; Xu et al. 2023; Wang et al. 2024). Regarding *qnrS*, despite the limited clinical impact of this resistance, its abundance may be considered as an indicator of fluoroquinolones (FQ) load in WWTPs as a consequence of its use (E. Castrignanò, Kannan, et al. 2020; A. Abramova et al. 2023). Additionally, the strong positive correlation between *intI1*, *sul1*, and *tetA*, highlights the involvement of *intI1* in HGT‐based spread of sulfonamides and tetracycline resistance (La Rosa et al. 2025). In this respect, the frequent localization of *sul1* in the 3′‐CS regions of *intI1* was already proven (Shamsizadeh et al. 2024; Su et al. 2012).

Among clinically relevant ARGs, *ermB* showed the highest mean absolute abundance (10^6 ^g.u./mL), consistent with the high consumption rate of MST, which are reported as the second most used classes of antimicrobial drugs in Italy (Agenzia Italiana del Farmaco AIFA 2025). Indeed, this gene showed significantly higher abundance in urban rather than rural areas, and its quantity positively correlated with the population size of the served geographical area. Interestingly, *ermB* was also found among the most abundant ARGs in sewage collected in Sicily, a region in Southern Italy (Magnano San Lio et al. 2025), and in other European countries, such as Poland (Zieliński et al. 2021). These wastewater‐based data agree with the positive correlation observed in EU/SEE countries among macrolide consumption and macrolide‐resistant *Streptococcus pneumoniae* prevalence (Agenzia Italiana del Farmaco AIFA 2025), confirming that the extensive use of MST drugs in human and veterinary medicine has led to the spread of this resistance. The abundance of *bla*_*CTX‐M*_, *bla*_*KPC*_, *vanA*, and *mecA* genes was much lower, ranging from 10^1^ to 10^4 ^g.c./mL, consistent with their role in resistance to antibiotics prevalently used in clinical settings. Among these genes, the most abundant one was *bla*_*CTX‐M*_, retrieved in similar quantities from all WWTPs. Third‐generation cephalosporins are the third most consumed antibiotic in Italy, and the prevalence of *E. coli* and *K. pneumoniae* resistant to third‐generation cephalosporins positively correlated with the antibiotic consumption (Agenzia Italiana del Farmaco AIFA 2025). Interestingly, while most ARGs were always found, *bla*_*KPC*_ and *vanA* could not be detected in some sewage samples, mainly coming from rural areas. Significantly higher abundances of both genes were indeed found in WWTPs serving urban areas, and a positive correlation with population size was observed. These results, besides being explainable with the higher population density of urban areas that may facilitate the transmission of resistant bacteria (Knight et al. 2025), can also be linked to the presence of hospitals, healthcare services, specialized medical centers, long‐term care facilities and other sites associated with high consumption of clinically used drugs, such as carbapenems and vancomycin. Indeed, two out of the three WWTPs serving urban areas, namely, WWTP2 and WWTP3, include wastewater from hospitals and, in fact, always showed the highest abundance of *bla*_*KPC*_ and *vanA* genes. The *mecA* gene, conferring resistance to methicillin, was the least abundant, in agreement with studies indicating this ARG, as well as the corresponding ARB (MRSA), as being mainly associated with hospital sewages instead of WWTPs serving the entire community (Volkmann et al. 2004; Meir‐Gruber et al. 2016). Correlation analysis indicated the co‐occurrence among some clinically relevant ARGs, suggesting the presence of multidrug‐resistant (MDR) bacterial strains. The observed positive correlation between *bla*_*CTX‐M*_ and *bla*_*KPC*_ as well as between *bla*_*CTX‐M*_ and *qnrS*, for instance, can be explained considering that these genes are often localized on mobile genetic elements, leading to association among β‐lactamase‐coding genes or with other resistances, such as those to fluoroquinolones (Briales et al. 2012; Dobiasova et al. 2013; Caltagirone et al. 2017). Similarly, co‐occurrence of *vanA* and *ermB* is coherent with the association among these resistances frequently observed in VRE strains (Toc et al. 2022; Mu et al. 2025). Interestingly, we found an increase in most ARG abundance with temperature, indicating a potential biological response whereby high temperatures, like suboptimal pH conditions, may favor the proliferation of microbial populations carrying ARGs and promote HGT (Magnano San Lio et al. 2025). This evidence may also suggest seasonal variations of AMR in wastewater, even if *ad‐hoc* and longer studies are necessary for a proper investigation of this matter. Indeed, while seasonality in the consumption of antibiotics is known, with peaks observed in winter months due to prescribing for respiratory tract infections, a recent literature review showed the occurrence of conflicting reports about temporal variation and seasonal influence on AMR in wastewater (Malcom and Bowes 2025).

Together with the assessment of AMR, we investigated the structure and composition of bacterial communities, which, overall, appeared quite homogenous among different WWTPs. This can be explained considering that, despite differences in terms of vocation and population sizes served, all samples originated from the same province and so from a relatively small geographical area. In addition, while *16S rRNA* amplicon sequencing based on short‐reads provided a satisfactory genus‐level classification, long‐read sequencing technologies could have achieved a higher resolution, that is, at species or strain level, allowing the discrimination of differences between closely related communities. In all samples, the most abundant phyla were represented by Pseudomonadota, Bacillota, Bacteroidota, Campylobacterota, and Actinomycetota, confirming the presence of a stable and shared core sewage bacterial community, as previously observed worldwide (Palanisamy et al. 2022; Rajput et al. 2025; Bonanno Ferraro et al. 2024).

Among the most abundant genera (i.e., those showing an average relative abundance higher than 1%), we sought to investigate the occurrence of pathogenic ARBs by comparing our results with the WHO BPPL 2024 (WHO 2024), which reports ARBs posing a high risk to human health. With this approach, we identified *Acinetobacter*, *Pseudomonas*, and *Streptococcus* as genera that could include priority ARBs, such as *A. baumannii* (carbapenem‐resistant), *P. aeruginosa* (carbapenem‐resistant), and *S. pneumoniae* (macrolide‐resistant), respectively (WHO 2024). *A. baumannii* and *P. aeruginosa*, two species belonging to the ESKAPEE group, together with other *Acinetobacter* and *Pseudomonas* pathogenic species, are often recovered from wastewater and show a similar pattern of resistance to that isolated from clinical samples (Hubeny et al. 2022; Paul et al. 2025). For instance, *A. baumannii* and *Acinetobacter junii* isolated from wastewater showed aminoglycoside and carbapenem resistances similarly to clinical isolates (Aguilar‐Vera et al. 2024; Castillo‐Ramírez et al. 2025). Likewise, the antibiotic susceptibility pattern of *P. aeruginosa* from wastewater aligned with epidemiological data (Monteagudo de Barros et al. 2024). These observations confirm the effectiveness of WBE in tracking human pathogens and defining their antibiotic‐resistance profile, providing useful insights for public health. However, even if *16S rRNA* amplicon sequencing indicated *Streptococcus* among the most abundant genera in wastewater (Numberger et al. 2019; Bonanno Ferraro et al. 2024), surveys comparing the resistance profiles of strains from clinical and wastewater samples are still lacking, probably due to the low load of these bacteria in sewages that limits the implementation of culture‐based surveillance approaches (Tiwari et al. 2022).

*Acinetobacter* and *Pseudomonas* decreased over time, while *Streptococcus* increased. These temporal dynamics may suggest a possible influence of both climatic and anthropogenic factors, as previously suggested (Becsei et al. 2024; Keer et al. 2025). Indeed, a strong positive correlation was observed between sample temperature and *Streptococcus*, while *Acinetobacter* and *Pseudomonas* showed a negative correlation with this parameter. Together, these data confirm the impact of climatic factors, such as atmospheric temperature, considering that our sampling campaign started at the end of the cold season (April) and proceeded throughout the hot season (September). Nevertheless, our 6‐month‐long study does not allow seasonality speculations, despite seasonal variations in wastewater bacterial communities, including genera listed in the BPPL 2024, having already been reported (Numberger et al. 2019; Hubeny et al. 2022; Bonanno Ferraro et al. 2024; Keer et al. 2025).

We found positive correlations of *Streptococcus* abundance with most of the ARGs studied. This is coherent with the well‐known rise of *Streptococcus* infections not responding to macrolides, as well as to tetracycline, penicillin, cephalosporins and fluoroquinolones (Gergova et al. 2024). This observation is particularly relevant given the increasing notification rate of invasive bacterial diseases caused by *S. pneumoniae* in Italy, including the province of Trento where our study was conducted (Giufré et al. 2025). However, the prevalence of macrolide‐resistant *S. pneumoniae* in the province of Trento is lower than in most of the Italian regions (Agenzia Italiana del Farmaco AIFA 2025). Interestingly, we did not observe a co‐occurrence of the *Acinetobacter* genus with any of the ARGs studied, despite bacteria belonging to the *A. baumannii–calcoaceticus* complex being often reported as MDR, as well as extensively drug resistant and pan‐drug resistant (Scoffone et al. 2025). This observation is consistent with data from the last National Antibiotic‐Resistance Surveillance showing that, despite a national average of 74.3% in 2024, no carbapenem‐resistant *Acinetobacter* clinical isolates were reported in the province of Trento during the last 5 years (Iacchini et al. 2025). Regarding *Pseudomonas*, a positive correlation occurred only with the *qnrS*, a plasmid‐associated gene conferring resistance to low levels of fluoroquinolones, despite the prevalence of FQ resistance in Pseudomonas, determined by transferable mechanisms, being usually low (López et al. 2022). An overall decrease in *P. aeruginosa* clinical isolates resistant to the most frequently used antibiotics to treat such infections, including fluoroquinolones and carbapenems, has been reported in Italy (Iacchini et al. 2025). The province of Trento showed the same trend, with the prevalence of carbapenem‐resistant *P. aeruginosa* decreasing from 14% in 2022 to 8.8% in 2024.

## 5 Conclusions

Wastewater surveillance conducted in this work demonstrated that both the prevalence of extended‐spectrum β‐lactamase‐producing *E. coli* and the abundance of clinically significant ARGs, specifically *ermB*, *bla*_*KPC*_, and *va*nA, were greater in urban areas compared with rural ones. NGS characterization of wastewater bacterial communities indicated the occurrence of abundant populations of *Acinetobacter*, *Pseudomonas*, and *Streptococcus*, genera that may include ARBs reported in the WHO BPPL. This work underscores the importance of implementing WBE studies across geographical areas characterized by different vocations, municipalities, and population sizes. This approach can, in fact, provide a deeper and more comprehensive understanding of population‐level trends and changes in AMR, contributing to more effective public health interventions.

## Author Contributions

**Maya Petricciuolo:** conceptualization, formal analysis, supervision, visualization, writing – original draft. **Agnese Carnevali:** data curation, investigation, validation, methodology. **Alessia Torboli:** formal analysis, investigation, writing – original draft. **Mattia Postinghel:** investigation, resources. **Alessia Guasticchi:** investigation, methodology, validation. **Paola Foladori:** supervision, writing – review and editing. **Maria Cadonna:** conceptualization, data curation, funding acquisition, resources. **Ermanno Federici:** conceptualization, supervision, funding acquisition, project administration, writing – review and editing, resources.

## Ethics Statement

The authors have nothing to report.

## Conflicts of Interest

None declared.

## Data Availability

The 16S rRNA amplicon sequencing data sets generated during the current study will be available upon acceptance in the NCBI Sequence Read Archive (SRA) repository under the BioProject ID PRJNA1402288. The data that support the findings of this study are openly available in NCBI SRA at https://www.ncbi.nlm.nih.gov/sra/PRJNA1402288, reference number PRJNA1402288.

## Appendix A

**Table A1:** List of collected wastewater samples. The table provides information about the collected sewage, including the sampling point, the sampling date, the flow rate (m^3^/day), and the physicochemical characteristics.

Sample_ID	WWTP	Sampling date	Flow (m^3^/h)	Temp (°C)	BOD5 (mg/L)	NH_4_ (mg/L)
S01‐WWTP1‐Apr	WWTP1	08/04/2024	1110	15.5	106	28.58
S02‐WWTP1‐May	WWTP1	13/05/2024	826	18.4	180	41.8
S03‐WWTP1‐June	WWTP1	19/06/2024	1310	19	169	26.23
S04‐WWTP1‐Jul	WWTP1	15/07/2024	1150	21.4	156	26.39
S05‐WWTP1‐Aug	WWTP1	19/08/2027	780	24.1	299	50.2
S06‐WWTP1‐Sept	WWTP1	09/09/2024	1400	22.4	179	26.87
S07‐WWTP2‐Apr	WWTP2	08/04/2024	665	19	309	34.1
S08‐WWTP2‐May	WWTP2	13/05/2024	613	16.2	239	36.3
S09‐WWTP2‐June	WWTP2	19/06/2024	950	19.6	202	19
S10‐WWTP2‐Jul	WWTP2	15/07/2024	772	21.7	164	20.8
S11‐WWTP2‐Aug	WWTP2	19/08/2027	486	25.6	218	33.6
S12‐WWTP2‐Sept	WWTP2	09/09/2024	892	24.8	188	20.1
S13‐WWTP3‐Apr	WWTP3	08/04/2024	569	15.6	168	31.4
S14‐WWTP3‐May	WWTP3	13/05/2024	674	17.6	300	34.3
S15‐WWTP3‐June	WWTP3	19/06/2024	690	17.8	226	27.5
S16‐WWTP3‐Jul	WWTP3	15/07/2024	583	20	200	25.87
S17‐WWTP3‐Aug	WWTP3	19/08/2027	455	21.4	285	40.1
S18‐WWTP3‐Sept	WWTP3	09/09/2024	662	20.9	273	31.3
S25‐WWTP4‐Apr	WWTP4	08/04/2024	47	13.9	222	26.4
S26‐WWTP4‐May	WWTP4	13/05/2024	34	16.4	365	17.8
S27‐WWTP4‐June	WWTP4	19/06/2024	49	17.9	127	23.3
S28‐WWTP4‐Jul	WWTP4	15/07/2024	52	20.1	125	26.4
S29‐WWTP4‐Aug	WWTP4	19/08/2027	50	21.9	350	10.2
S30‐WWTP4‐Sept	WWTP4	09/09/2024	64	21.6	201	21.1
S37‐WWTP5‐Apr	WWTP5	08/04/2024	21	9.4	164	26.9
S38‐WWTP5‐May	WWTP5	13/05/2024	15	12.2	303	14.1
S39‐WWTP5‐June	WWTP5	19/06/2024	31	17.8	128	20.5
S40‐WWTP5‐Jul	WWTP5	15/07/2024	29	20	163	15.1
S41‐WWTP5‐Aug	WWTP5	19/08/2027	19	22.8	170	15.2
S42‐WWTP5‐Sept	WWTP5	09/09/2024	18	22.3	261	26.3
S31‐WWTP6‐Apr	WWTP6	08/04/2024	38	12.8	138	13.1
S32‐WWTP6‐May	WWTP6	13/05/2024	25	15.2	199	8.5
S33‐WWTP6‐June	WWTP6	19/06/2024	44	16.1	187	13.2
S34‐WWTP6‐Jul	WWTP6	15/07/2024	44	17.8	75	8.9
S35‐WWTP6‐Aug	WWTP6	19/08/2027	28	20.3	215	13.3
S36‐WWTP6‐Sept	WWTP6	09/09/2024	42	19.9	194	12.1
S19‐WWTP7‐Apr	WWTP7	08/04/2024	5.4	12	356	39
S20‐WWTP7‐May	WWTP7	13/05/2024	4	15.2	364	16.4
S21‐WWTP7‐June	WWTP7	19/06/2024	8	18.3	294	30
S22‐WWTP7‐Jul	WWTP7	15/07/2024	4.8	22.2	406	44.6
S23‐WWTP7‐Aug	WWTP7	19/08/2027	7.5	23.6	430	19.6
S24‐WWTP7‐Sept	WWTP7	09/09/2024	9	22.3	750	38.9

Abbreviations: BOD, biochemical oxygen demand; WWTP, wastewater treatment plant.

**Table A2:** List of qPCR primers and main PCR conditions.

Target gene	Primer name	Primer sequence	Primer final concentration (µM)	qPCR reaction conditions	Amplicon size (bp)	Reference
*16S rRNA*	357F	CCTACGGGAGGCAGCAG	0.05	95°C 600″; 95°C 10″–55°C 10″–72°C 10″ (45×)	194	Muyzer et al. (1993)
	518R	ATTACCGCGGCTGCTGG	0.9			
*sul1*	*sulI*‐FW	CGCACCGGAAACATCGCTGCAC	0.3	95°C 60″; 95°C 15″–60°C 30″ (45×)	162	Pei et al. (2006)
	*sulI*‐RV	TGAAGTTCCGCCGCAAGGCTCG				
*tetA*	*tetA*_f	GCTACATCCTGCTTGCCTTC	0.3	95°C 60″; 95°C 15″–60°C 30″ (45×)	210	Ng et al. (2001)
	*tetA*_r	CATAGATCGCCGTGAAGAGG				
*intI1*	*intI1*‐LC5 F	GATCGGTCGAATGCGTGT	0.5	95°C 60″; 95°C 15″–60°C 30″ (45×)	196	Barraud et al. (2010)
	*intI1*‐LC1 R	GCCTTGATGTTACCCGAGAG				
*qnrS*	*qnrS*rtF11	GACGTGCTAACTTGCGTGAT	0.2	95°C 60″; 95°C 15″–60°C 30″ (45×)	118	Marti and Balcázar (2013)
	*qnrS*rtR11	TGGCATTGTTGGAAACTTG				
*ermB*	*erm(B)*F	CCGAACACTAGGGTTGCTC	0.6	95°C 60″; 95°C 15″–60°C 30″ (45×)	139	Di Cesare et al. (2013)
	*erm(B)*R	ATCTGGAACATCTGTGGTATG				

Abbreviation: qPCR, quantitative polymerase chain reaction.

**Table A3:** List of dPCR primers and main PCR conditions.

Target gene	Primer name	Primer sequence	Primer final concentration (µM)	dPCR reaction conditions	Amplicon size (bp)	Primer reference
*bla*_*CTX‐M*_	RT*CTXM*‐F	CTATGGCACCACCAACGATA	0.4	95°C 2′; 95°C 15″–60°C 15″–72°C 15″ (45×); 40°C 5′	103	Marti Serrano. (2014)
	RT*CTXM*‐R	ACGGCTTTCTGCCTTAGGTT				
*bla*_*KPC*_	*Kpc*‐rtF	CAGCTCATTCAAGGGCTTTC	0.4	95°C 2′; 95°C 15″–60°C 15″–72°C 15″ (45×); 40°C 5′	196	Subirats. et al. (2017)
	*Kpc*‐rtR	GGCGGCGTTATCACTGTATT				
*mecA*	*mecA*1FP	CGCAACGTTCAATTTAATTTTGTTAA	0.4	95°C 2′; 95°C 15″–60°C 15″–72°C 15″ (45×); 40°C 5′	92	Volkmann et al. (2004)
	*mecA*1RP	TGGTCTTTCTGCATTCCTGGA				

Abbreviation: dPCR, digital polymerase chain reaction.

## Appendix B

**Table B1:** Summary of results obtained from the enumeration of total *Escherichia coli*. ESBL*‐Ec* and percentage of ESBL‐*Ec* over total *E. coli* in all the selected WWTPs. For each WWTP and for each parameter, the minimum and maximum loads are reported together with the median value. the interquartile range (IQR) and the mean value with standard deviation (sd).

	No. of samples^a^	Parameter	Minimum	Maximum	Median	IQR	Mean (sd)
WWTP1	6	Total *E. coli*/100 mL	3.30 × 10^5^	3.65 × 10^6^	1.68 × 10^6^	1.09 × 10^6^	1.65 × 10^6^ (1.19 × 10^6^)
		ESBL‐*Ec*/100 mL	2.15 × 10^4^	2.30 × 10^5^	5.03 × 10^4^	4.21 × 10^4^	7.45 × 10^4^ (7.88 × 10^4^)
		ESBL‐*Ec* over total *E. coli* (%)	2.00	11.50	3.65	3.23	5 (3.58)
WWTP2	5	Total *E. coli*/100 mL	7.60 × 10^4^	3.82 × 10^6^	2.27 × 10^6^	1.27 × 10^6^	2.32 × 10^6^ (1.45 × 10^6^)
		ESBL‐*Ec*/100 mL	8.09 × 10^3^	2.43 × 10^5^	1.00 × 10^5^	5.45 × 10^4^	1.20 × 10^5^ (8.57 × 10^4^)
		ESBL‐*Ec* over total *E. coli* (%)	2.90	11.70	6.34	1.91	6.49 (3.28)
WWTP3	5	Total *E. coli*/100 mL	1.50 × 10^6^	1.09 × 10^7^	3.11 × 10^6^	4.07 × 10^6^	4.59 × 10^6^ (3.92 × 10^6^)
		ESBL‐*Ec*/100 mL	1.20 × 10^5^	3.81 × 10^5^	1.87 × 10^5^	1.26 × 10^5^	2.23 × 10^5^ (1.06 × 10^5^)
		ESBL‐*Ec* over total *E. coli* (%)	3.50	8.90	6.01	3.20	6.24 (2.23)
WWTP4	6	Total *E. coli*/100 mL	1.37 × 10^6^	9.50 × 10^6^	2.91 × 10^6^	1.86 × 10^6^	3.64 × 10^6^ (3.02 × 10^6^)
		ESBL‐*Ec*/100 mL	4.65 × 10^4^	1.43 × 10^5^	1.16 × 10^5^	5.05 × 10^4^	1.08 × 10^5^ (3.92 × 10^4^)
		ESBL‐*Ec* over total *E. coli* (%)	1.50	10.00	3.51	4.08	4.45 (3.31)
WWTP5	6	Total *E. coli*/100 mL	4.25 × 10^5^	1.42 × 10^7^	4.95 × 10^6^	7.46 × 10^6^	6.09 × 10^6^ (5.39 × 10^6^)
		ESBL‐*Ec*/100 mL	1.70 × 10^4^	6.05 × 10^5^	9.85 × 10^4^	2.17 × 10^5^	2.10 × 10^5^ (2.28 × 10^5^)
		ESBL‐*Ec* over total *E. coli* (%)	1.68	5.6	3.73	1.49	3.59 (1.38)
WWTP6	6	Total *E. coli*/100 mL	9.85 × 10^5^	4.13 × 10^6^	2.87 × 10^6^	1.43 × 10^6^	2.87 × 10^6^ (1.18 × 10^6^)
		ESBL‐*Ec*/100 mL	1.45 × 10^4^	1.17 × 10^5^	6.85 × 10^4^	2.84 × 10^4^	6.55 × 10^4^ (3.45 × 10^4^)
		ESBL‐*Ec* over total *E. coli* (%)	1.50	3.40	1.90	0.82	2.22 (0.73)
WWTP7	5	Total *E. coli*/100 mL	9.80 × 10^5^	3.63 × 10^6^	2.75 × 10^6^	8.5 × 10^5^	2.60 × 10^6^ (1.02 × 10^6^)
		ESBL‐*Ec*/100 mL	3.20 × 10^4^	1.79 × 10^5^	1.35 × 10^5^	4.3 × 10^4^	1.16 × 10^5^ (5.55 × 10^4^)
		ESBL‐*Ec* over total *E. coli* (%)	2.60	7.45	4.25	1.60	4.50 (1.87)

Abbreviations: ESBL, extended‐spectrum β‐lactamases; WWTP, wastewater treatment plant.

a A total of six samples for each sampling point were collected and analyzed. Samples identified as outliers were excluded from data analysis.

**Table B2:** Differential abundance analysis. The table reports the differences in the relative abundance of genera with prevalence exceeding 1% between samples grouped by geographical area (urban vs. rural). Results with an adjusted (Benjamini and Hochberg method) *p* ≤ 0.05 (padj) and log2fold > 1 were considered significant (*).

Genus	baseMean	log2FoldChange	lfcSE	Stat	padj
*Flavobacterium*	2872.13	−0.82	0.37	−2.2	0.09
*Cloacibacterium*	3080.49	0.59	0.40	1.46	0.162
*Arcobacter*	3748.45	0.50	0.29	1.75	0.125
*Simplicispira*	903.49	−0.91	0.32	−2.81	0.031
*Aeromonas*	5166.14	−0.542	0.32	−1.71	0.125
*Acinetobacter*	19,396.34	−0.37	0.19	−1.94	0.125
*Enhydrobacter*	1512.12	0.66	0.41	−1.6	0.137
*Pseudomonas*	1325.12	−0.07	0.26	−0.26	0.789
*Streptococcus (*)*	921.86	1.2	0.44	2.74	0.031
*Trichococcus*	1640.10	−0.65	0.35	−1.87	0.125

*Note:* Log2 fold change standard error (lfcSE) values are also reported.

**Table B3:** Relative abundance of genera listed in the WHO Bacterial Priority Pathogens List 2024 (BPPL 2024). This table reports the relative abundance of the genera that exceeded 1% of prevalence and were included in the BBLP 2024, that is, *Acinetobacter*, *Pseudomonas*, and *Streptococcus* in all WWTPs at each sampling month.

	April	May	June	July	August	September
WWTP1						
*Acinetobacter* (%)	28.51	26.03	23.02	18.89	6.49	11.35
*Pseudomonas* (%)	1.83	2.82	1.10	0.92	0.60	0.76
*Streptococcus* (%)	0.15	0.20	0.39	1.38	2.11	2.63
WWTP2						
*Acinetobacter* (%)	35.72	27.75	28.60	35.73	20.02	23.22
*Pseudomonas* (%)	6.71	3.02	2.29	1.91	2.40	0.92
*Streptococcus* (%)	0.58	0.68	0.80	1.73	6.73	3.28
WWTP3						
*Acinetobacter* (%)	41.80	28.35	34.52	33.87	22.43	27.81
*Pseudomonas* (%)	2.17	2.58	2.25	1.63	1.15	2.60
*Streptococcus* (%)	0.31	0.33	0.76	1.41	3.94	2.59
WWTP4						
*Acinetobacter* (%)	41.00	30.45	29.98	39.42	30.15	27.15
*Pseudomonas* (%)	2.89	3.48	3.09	1.23	0.89	1.45
*Streptococcus* (%)	0.19	0.15	0.18	0.74	1.20	1.53
WWTP5						
*Acinetobacter* (%)	24.28	14.80	6.51	20.76	10.06	11.72
*Pseudomonas* (%)	2.36	1.62	1.05	2.63	1.57	0.72
*Streptococcus* (%)	0.25	0.46	0.19	0.83	1.03	0.88
WWTP6						
*Acinetobacter* (%)	40.99	28.95	27.13	39.59	28.45	26.30
*Pseudomonas* (%)	2.47	3.14	1.55	1.72	0.67	0.86
*Streptococcus* (%)	0.17	0.16	0.20	0.31	1.60	1.38
WWTP7						
*Acinetobacter* (%)	47.01	18.57	34.53	14.33	20.50	19.27
*Pseudomonas* (%)	4.97	2.12	1.10	0.19	0.73	0.29
*Streptococcus* (%)	0.28	0.40	0.34	0.87	1.14	0.86

Abbreviation: WWTP, wastewater treatment plant.
